# Characterization and variation of the rhizosphere fungal community structure of cultivated tetraploid cotton

**DOI:** 10.1371/journal.pone.0207903

**Published:** 2019-10-18

**Authors:** Qinghua Qiao, Jingxia Zhang, Changle Ma, Furong Wang, Yu Chen, Chuanyun Zhang, Hui Zhang, Jun Zhang

**Affiliations:** 1 Key Laboratory of Plant Stress Research, College of Life Sciences, Shandong Normal University, Jinan, China; 2 Key Laboratory of Cotton Breeding and Cultivation in Huang-Huai-Hai Plain, Ministry of Agriculture, Cotton Research Center of Shandong Academy of Agricultural Sciences, Jinan, China; Tallinn University of Technology, ESTONIA

## Abstract

Rhizosphere fungal communities exert important influencing forces on plant growth and health. However, information on the dynamics of the rhizosphere fungal community structure of the worldwide economic crop cotton (*Gossypium* spp.) is limited. In the present study, next-generation sequencing of nuclear ribosomal internal transcribed spacer-1 (ITS1) was performed to characterize the rhizosphere fungal communities of *G*. *hirsutum* cv. TM-1 (upland cotton) and *G*. *barbadense* cv. Hai 7124 (island cotton). The plants were grown in field soil (FS) that had been continuously cropped with cotton and nutrient-rich soil (NS) that had not been cropped. The fungal species richness, diversity, and community composition were analyzed and compared among the soil resources, cotton genotypes, and developmental stages. We found that the fungal community structures were different between the rhizosphere and bulk soil and the difference were significantly varied between FS and NS. Our results suggested that cotton rhizosphere fungal community structure variation may have been primarily influenced by the interaction of cotton roots with different soil resources. We also found that the community composition of the cotton rhizosphere fungi varied significantly during different developmental stages. In addition, we observed fungi that was enriched or depleted at certain developmental stages and genotypes in FS and NS, and these insights can lay a foundation for deep research into the dynamics of pathogenic fungi and nutrient absorption of cotton roots. This research illustrates the characteristics of the cotton rhizosphere fungal communities and provides important information for understanding the potential influences of rhizosphere fungal communities on cotton growth and health.

## Introduction

Soil microorganisms play a key role in agricultural ecosystem. The importance of the mutual influence between microbial communities and agronomic practices is increasingly being recognized. The rhizosphere is the soil area that adjacent to plant roots, in which the interactions between soil microorganism and plant roots are very intense. Plants play important roles on rhizosphere microbiome assembly and functions [1]. The composition of rhizosphere microbial communities is affected by the soil, plant developmental stage, and many other factors [2–6]. Rhizosphere microorganisms are considered pivotal for plant health and growth due to their involvement in such key processes as the formation of the root architecture [7]; formation of soil characteristics [8]; decomposition of organic matter [9, 10]; decomposition and removal of toxins [11, 12]; defense against plant pathogenic microorganisms and pests [7, 13]; and cycling of carbon [14], nitrogen, phosphorus, and sulfur [15–18].

Rhizosphere fungi are critical component of the rhizosphere microbial communities, and play an important role in plant growth and health. In turn, plants largely control rhizosphere fungi through the production of carbon- and energy-rich compounds and bioactive phytochemicals [19]. Some of the beneficial fungi are directly involved in the cycling of nutrients and function as an essential link for soil nutrient availability [20–23]. Some fungi are known for having biocontrol activity against pathogenic microorganisms [23, 24]. These fungi positively influence plant productivity by enhancing plant growth. However, certain rhizosphere fungi can negatively influence plant productivity by infecting roots and causing serious disease. For example, *Fusarium graminearum* can cause stalk rot disease of maize [25], *Verticillium nonalfalfae* could cause *Verticillium* wilt on tree-of-heaven [26], and *Macrophomina phaseolina* can cause dry root rot disease [27].

Cotton (*Gossypium* spp.) is the most important cash crop and is widely grown to produce both natural fibers and cotton seed soil. Cotton production is threatened by soil-borne plant pathogens, such as *Rhizoctonia* spp. [28], *Fusarium moniliforme* [29], *Alternaria alternata* [30], and *Verticillium dahliae* [31]. Understanding the dynamics of the rhizosphere fungal community structure of cotton during different developmental stages will not only provide basic information on the dynamics of the cotton rhizosphere fungal community structure but also help lay a foundation for understanding the mutual influence between rhizosphere fungal communities and the plant health of cotton. Knox *et al*. showed that rhizosphere microbial diversity in cotton is significantly influenced by the cultivar type in the field [32]. However, systematic studies on the rhizosphere fungal community structure of cultivated tetraploid cotton are still lacking.

This study characterized the rhizosphere fungal community dynamics across cotton developmental stage growth using two cotton cultivars in two different types of soil. Our work lays the foundation for cotton rhizosphere fungal community research and provides insights into the structure of rhizosphere fungal communities and the potential roles on cotton growth and health in the agricultural production.

## Materials and methods

### Plants and soil

Two cultivars of cultivated allotetraploid *Gossypium* species, *G*. *hirsutum* cv. TM-1 (upland cotton) and *G*. *barbadense* cv. Hai 7124 (island cotton with higher disease resistance than upland cotton) were planted in field soil (FS) and nutrient-rich soil (NS) as described in detail.

The soil samples were prepared according to the following method. The FS was obtained from 15 to 30 cm below the soil surface in a field that has been continuously planted with cotton for several decades at the Experiment Station of Cotton Research Center of Shandong Academy of Agricultural Sciences (Linqing County, Shandong Province, 36°81′N, and 115.71°13′E). The NS is a type of horticultural medium composed of leaf mold, plant ash, bone meal, river sand, etc., and it was obtained from Feng Yuan Science and Technology Company (Jinan, China). All visible biota (e.g., weeds, twigs, worms, and insects) were removed, and the soil was then crushed and sifted through a sterile 2 mm sieve. Because the sieved soil drained poorly and was difficult to sample, we mixed sterile sand into the treatment soils at a soil: sand ratio of 2:1 following Lundberg *et al*. [33]. The characteristics of the soils were listed in S1 Materials and methods.

All plants were grown under the same environmental conditions. Each type of soil was placed in large pots and divided into two groups. For the rhizosphere sample, cotton was grown in the soil; for the bulk soil sample, cotton was not planted. Cotton seeds were surface sterilized and germinated, then transplanted into the soils and raised in a tissue culture room at 28°C. After germination, the cotton seedlings were transplanted into the various soils and seedling were raised in a tissue culture room at 28 °C. Move the seedlings to a greenhouse when it developed a second real leaf.

### Sampling of the rhizosphere and bulk soil

Invert the pot to remove the soil and plant. Then shake the plant gently to remove the soil that did not adhere to the root surface. Soil that tightly adhered to the root surface and was not easily shaken from the root was the rhizosphere soil. Place the roots with attached soil in a sterile flask with 50 ml of sterile buffered phosphate saline solution and stirred vigorously with sterile forceps to clean all the soil from the root surfaces. Then remove the cleaned roots and centrifugated the fluid for 15 min at 10,000 rpm. Discard the supernatant and the soil fraction was quickly frozen using liquid nitrogen, then stored at -80 °C. Bulk soil samples were collected from unplanted pots from ~10 cm below the soil surface. Three biological replicates of each treatment were performed. The rhizosphere and bulk soil samples of the two cultivars in the FS and NS were collected at three developmental stages. In total, fifty-four samples were collected. Three biological replicates were performed for each treatment. Detailed information about the plant management and sampling of the cotton rhizosphere and bulk soil were described in our previous report [34].

### DNA extraction and detection

The DNA from each soil sample was extracted using the Omega D5625-02 Soil DNA Kit (Omega Biotek Inc., Norcross, GA, USA) as per the manufacturer’s instructions. The DNA concentration and integrity were detected by a microplate reader (Qubit 3.0 Fluorometer; Thermo Fisher Scientific, Waltham, MA, USA) and agarose gel electrophoresis (PowerPac Basic164-5050 and Sub-Cell 96, Bio-Rad Laboratories, Hercules, CA, USA). DNA information for each sample is listed in the S1 materials and methods.

### Preparation of libraries and sequencing

All suitable DNA samples were submitted to BGI Tech Solutions Co., Ltd. (Shenzhen, China) to construct a sequencing library. ITS1 (the internal transcribed spacer 1) (primer: ITS1-F:CTTGGTCATTTAGAGGAAGTAA; ITS1-R:GCTGCGTTCTTCATCGATGC) amplicon libraries was generated with DNA from 54 soil samples and sequenced using the Illumina MiSeq platform (Illumina, San Diego, CA, USA). Operational procedures were carried out in accordance with company SOP (Standard Operating Procedure). Sequence data were treated following the pipeline developed before [35]. Further details on the subsequent bioinformatics analysis of the sequencing data are listed in the S1 Materials and methods.

### Data analysis

#### OTU Venn diagram

The presence or absence of operational taxonomic units (OTUs) was determined for each soil sample, and the common and specific OTU IDs were summarized. A Venn diagram was constructed using the package VennDiagram in R (v 3.0.3).

#### Species annotation

The tag numbers of each phylum in the different soil samples were summarized in a histogram, and all data were used to construct a histogram using R.

#### α-diversity analysis

The species accumulation curves of the observed species (Sobs), Chao, Abundance Based Coverage Estimator (ACE), Shannon, and Simpson indices were calculated using the software Mothur (v 1.31.2). The calculation formula of each index can be found at http://www.mothur.org/wiki/Calculators. A rarefaction curve was drawn by the software R (v3.0.3) based on the expected value of α-diversity. First, the OTU numbers were calculated based on extracted tags (in multiples of 1000), and then, the rarefaction curve was drawn using the α-diversity indices calculated with extracted tags. The results are shown in the S1–S5 Figs. With the increase of OUT number, the trend of rare factions was tended to be stable, indicating the sequencing data was adequate.

#### β-diversity analysis

β-diversity was analyzed using the software QIIME (v 1.80). Normalization was performed to control for sequencing depth differences in different samples. The sequences were extracted randomly according to the minimum sequence number of all samples to generate a new ‘OTU table biom’ file. Then, the β-diversity distance was calculated based on the ‘OTU table biom’ file. The β-diversity heat map was drawn by the ‘aheatmap’ function in the ‘NMF’ package of R.

#### Contribution of each factor

The Bray-Curtis dissimilarity analysis and the information entropy method were used to measure the contribution of the different factors to variability between samples. We then conducted an analysis of variance by the function aov in the R package. Interactions between each of the two factors were considered. For each factor, the contribution rate to fungal community variance was calculated as the mean square of the factor divided by the sum of the mean square of all factors.

## Results

The fungal communities were characterized by next-generation sequencing of nuclear ribosomal ITS1. A total of 5,032,042 high-quality reads were obtained with a median read count of 93,186 per sample (range: 51,752–244,354) (S1 Table). The high-quality reads were clustered into 1,298 microbial OTUs at 97% similarity after the removal of OTUs that were unassigned or not assigned to the target species.

### Fungal communities in the bulk soils of FS and NS

Ascomycota, Basidiomycota, and Zygomycota were the most common fungal phyla in both the continuously cropped FS and NS treatments, and they accounted for 59.01–95.81% of all fungal communities (S2 Table; S6 Fig). Excluding unclassified orders (19.39–60.96% of total fungal communities) in both soils, Eurotiales and Hypocreales were dominant in Ascomycota, and Mortierellales was dominant in Zygomycota. The dominant orders of Basidiomycota in the FS were Cystofilobasidiales and Sporidiobolales, whereas the dominant orders in the NS were Thelephorales and Agaricales.

The differences in the fungal communities between the FS and NS soils at the genus level were significant. The relative abundance of certain fungal genera, such as *Penicillium*, *Gliomastix*, and *Engyodontium*, was significantly lower in the FS than NS (*P* < 0.05), whereas the relative abundance of certain fungal genera, such as *Pseudozyma*, *Panaeolus*, and *Lecanicillium* in the FS was slightly but not significantly higher than that in the NS (S2 Table).

### Fungal communities of the cotton rhizosphere in the FS and NS

**Fig 1:**
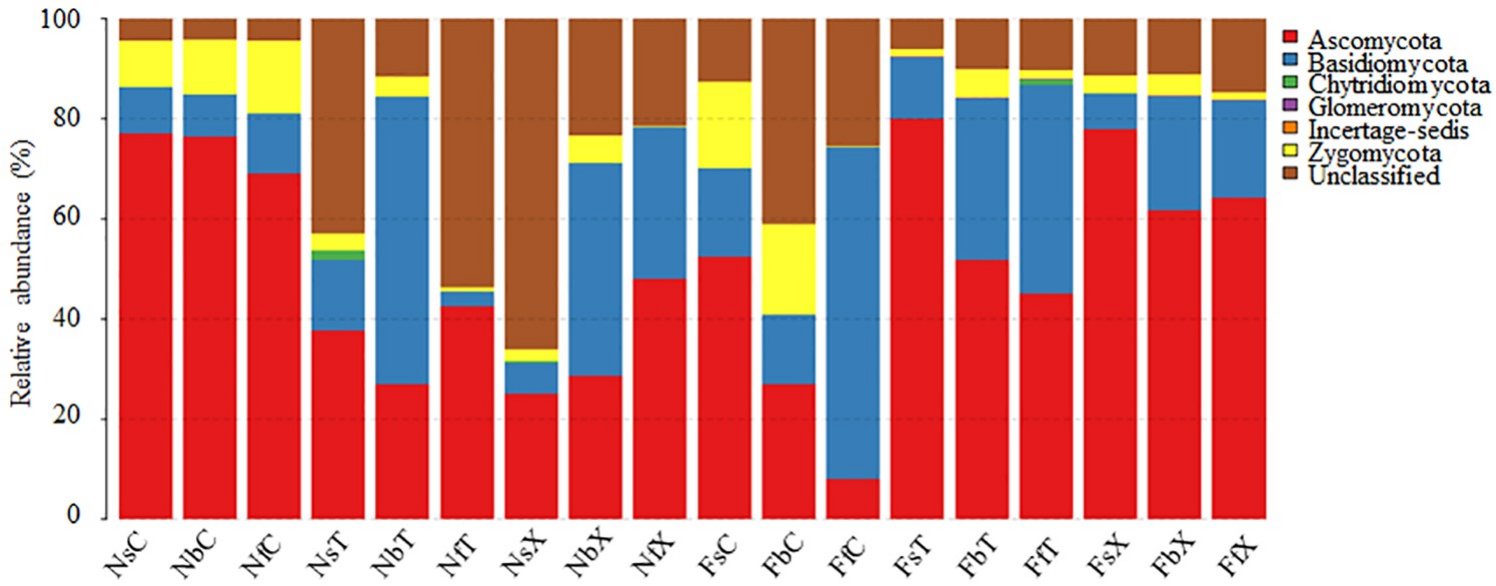
Relative abundance of the fungal community in all treatments. Two types of soils: nutrient-rich soil (N) and continuous cropping field soil (F). Three cotton plant developmental stages: seedling stage (s), budding stage (b), and flowering stage (f). Two cultivated species: upland cotton (*G*. *hirsutum* L. cv TM-1) (T) and sea island cotton (*G*. *barbadense* L. cv Hai 7124) (X) and control pots (C) that lacked cotton plants. Each sample was labeled by a three-letter code, such as NsT, which indicates seedlings of Sea Island cotton grown in nutrient-rich soil.

Ascomycota, Basidiomycota, and Zygomycota were the dominant phyla in the rhizosphere fungal communities and accounted for approximately 33.45–88.51% of the total fungal communities in the NS (11.48–66.15% were unclassified) and 85.18–93.88% of the total fungal communities in the FS (6.03–14.65% were unclassified) (Fig 1; S3 Table). Ascomycota was negatively selected in the rhizosphere in the NS but was enriched in the rhizosphere in the FS (Fig 1; S2–S4 Tables). The dominant orders of Ascomycota and Zygomycota in the rhizosphere were the same as those in the bulk soil (S3 Table). However, the dominant orders of Basidiomycota in the bulk soil from the FS rhizosphere samples were Agaricales and Auriculariales, whereas the dominant orders in the bulk soil from the NS rhizosphere samples were Sporidiobolales and Agaricales (S3 Table).

**Fig 2:**
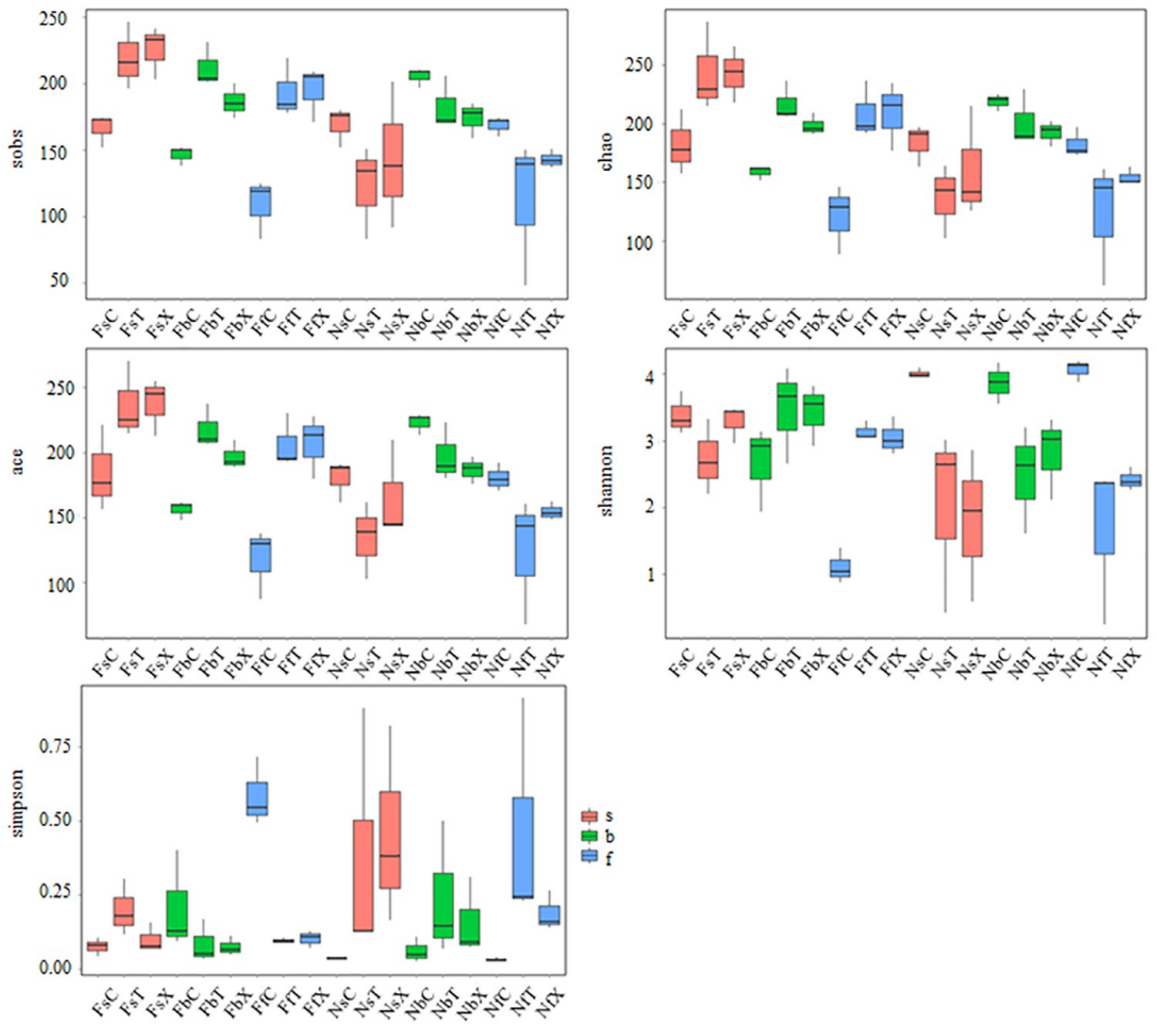
α-diversity of the rhizosphere fungi. From left to right and top to bottom, the box plots show the Sob, Chao, ACE, Shannon, and Simpson indices.

The number of OTUs in the FS rhizosphere (205.33 ± 22.47) was higher than that in the FS bulk soil (140.67 ± 28.61), whereas the number of OTUs in the NS rhizosphere (146.44 ± 40.22), was lower than that in the NS bulk soil (181.11 ± 20.37) (S5 Table). The α-diversity of fungi was significantly higher in the FS rhizosphere than in the FS bulk soil (*P* < 0.05); however, it was significantly lower in the NS rhizosphere than in the corresponding bulk soil (*P* < 0.05). The bulk soil α-diversity of fungi was higher in the NS than in the FS (*P* < 0.05), whereas the rhizosphere fungal α-diversity was lower in the NS than in the FS (*P* < 0.05; Fig 2; S5 Table).

Fungal genera were enriched or negatively selected in the rhizosphere compared with the corresponding bulk soil (S6 and S7 Tables). For example, in the NS, the relative abundance of *Mortierella*, *Gliomastix*, and *Engyodontium* was significantly higher in the bulk soil compared with the rhizosphere soil, where it was much lower or almost undetectable (*P*<0.05; S8 Table). In contrast, the relative abundance of *Rhodosporidium* and *Trichoderma* in the NS rhizosphere soil was higher than that in the respective bulk soil, where it was lower or almost undetectable (*P*<0.05; S8 Table). In the FS, the relative abundance of *Mortierella*, *Guehomyces*, and *Fusarium* was higher in the bulk soil than in the rhizosphere soil, where it was lower or undetectable (*P*>0.05; S9 Table). The relative abundance of *Penicillium*, *Alternaria*, and *Preussia* was higher in the FS rhizosphere soil than in the bulk soil, where these genera were almost undetectable (*P*<0.05; S9 Table). The abundance of other rhizosphere fungal genera was highly variable and differed between soils. Comparisons of the fungal genera whose relative abundance changed inversely in different soils between the rhizosphere and corresponding bulk soil are listed in Table 1.

**Table 1:** Fungal genera that were affected inversely by cotton roots in the two soil resources with differences in relative abundance between the bulk soil and rhizosphere soil > 0.15% (*P* < 0.05).

Developmental stage	Genera	Relative abundance in FS (%)		Relative abundance in NS (%)	
		Control	Rhizosphere	Control	Rhizosphere
**Seeding**	*Gliomastix*	0.00±0.00	0.42±0.28	2.68±0.89	0.00±0.00
	*Retroconis*	0.00±0.00	0.17±0.07	1.59±0.21	0.00±0.00
**Budding**	*Gibberella*	0.00±0.00	0.28±0.17	0.89±0.24	0.21±0.08
	*Gliomastix*	0.00±0.00	0.65±0.48	2.95±0.50	0.00±0.00
	*Penicillium*	0.40±0.06	14.13±4.99	9.94±1.89	3.84±1.99
	*Retroconis*	0.00±0.00	0.34±0.12	1.97±0.39	0.00±0.00
	*Guehomyces*	4.56±1.16	0.00±0.00	0.00±0.00	0.88±0.34
**Flowering**	*Engyodontium*	0.01±0.00	0.41±0.20	2.52±0.96	0.07±0.00
	*Gliomastix*	0.00±0.00	0.16±0.09	2.91±0.71	0.00±0.00
	*Mortierella*	0.17±0.07	2.03±0.88	16.2±4.56	1.14±0.07
	*Penicillium*	0.50±0.17	2.61±1.30	12.78±1.58	5.47±0.96

### Variation in rhizosphere fungal communities during development in FS

In the FS, the number of stage-specific OTUs was highest in the seedling stage and decreased gradually through development: upland cotton (T): 90 (seedling stage), 76 (budding stage), and 83 (flowering stage); and island cotton (X): 121 (seedling stage), 53 (budding stage), and 48 (flowering stage). And the number of overlapping OTUs in the seedling and budding stages was higher than that in the budding and flowering stages (S7 Fig).

An analysis of α-diversity indicated that in the FS, the Sobs, Chao, and ACE indices were higher in the cotton rhizosphere fungal communities during all three developmental stages compared with that in the bulk soil (*P* < 0.05). The three indexes all highest in seedling stage, and pretend different change of different cotton genotype. In the rhizosphere soil of TM-1, the three indexes were decreased gradually from the seedling stage to the flowering stage, but not significantly. And in island cotton rhizosphere soil, the three indexes were decreased significantly from seedling stage to budding stage (*P*<0.05), increased from budding stage to flowering stage (*P*>0.05; Fig 2; S5 Table). Analysis of Shannon and Simpson indices indicated that α-diversity were highest in budding stage.

### Variation in rhizosphere fungal communities during development in NS

In the NS, the number of stage-specific OTUs was highest in the budding stage (T: 71, 139, 85; X: 112, 138, 82). And overlapping OTUs of the seedling and budding stages was richer than that in the budding and flowering stages. The number of overlapping OTUs in all three developmental stages was higher in the FS than in the NS (S2 Fig). α-diversity analysis indicated that in the NS, the rhizosphere harbored a fungal community of higher α-diversity than bulk soil. The Sobs, Chao, and ACE indices of rhizosphere soil fungal communities were highest in budding stage (*P* < 0.05). But Shannon and Simpson indices have no significant difference between difference developmental stages, which indicated that the evenness of budding stage was lower than seeding and flowering stages.

### Variation in rhizosphere fungal communities in general level

Each developmental stage presented dominant fungal genera with a high relative abundance. We determined the genera that had high relative abundance (relative abundance > 0.5) in the different developmental stages. In the rhizosphere soils, *Penicillium*, *Fusarium*, and *Mortierella* presented a higher relative abundance in all three developmental stages in the FS and *Penicillium*, *Fusarium*, and *Talaromyces* presented a higher relative abundance in all three developmental stages in the NS. In addition, each developmental stage harbored the specific dominant rhizosphere fungal genera (S10 Table). The number of dominant genera was highest in the budding stage.

We also analyzed how the fungal community was affected by the presence of cotton. A large change was defined as a difference in the relative abundance between the rhizosphere and bulk soil at >1 or <-1. We defined a genus for which relative abundance was greater in the rhizosphere soil than in the bulk soil as an enriched fungal genus (EFG) and a genus for which relative abundance was lower in the rhizosphere soil than in the bulk soil as a depleted fungal genus (DFG). In FS, *Penicillium* was common EFG of TM-1 and Hai 7124 in seedling and flowering stage. *Guehomyces* were common DFG of TM-1 and Hai 7124 in seedling and budding stage. *Mrakia* were common DFG of TM-1 and Hai 7124 in seedling stage and *Hypholoma* were common DFG of TM-1 and Hai 7124 in flowering stage. In addition, each genotype has special EFG or DFG: *Preussia* were special EFG of TM-1in seedling stage; *Penicillium* were special EFG of Hai 7124 in budding stage; *Mortierella* were special EFG of Hai 7124 in flowering stage. In NS, common genus of TM-1 and Hai 7124, which relative abundance effected by cotton root significantly, were all DFG. For example, *Verticillium*, *Retroconis*, *Gliomastix*, *Gibberella* and *Metarhizium* were common DFG in seedling stage, *Verticillium*, *Retroconis*, *Gliomastix*, *Engyodontium* and *Mortierella* were common DFG in budding stage, *Retroconis*, *Gliomastix*, *Engyodontium* and *Mortierella* were common DFG in budding stage. In addition, *Penicillium* were special DFG of Hai 7124 in seedling stage and budding stage; *Mortierella* were special DFG of Hai 7124 in seedling stage; *Gibberella* and *Rhodosporidium* were special DFG of Hai 7124 in flowering stage (S10 Table).

### Contribution of each factor to the variation of cotton rhizosphere fungal community

**Fig 3:**
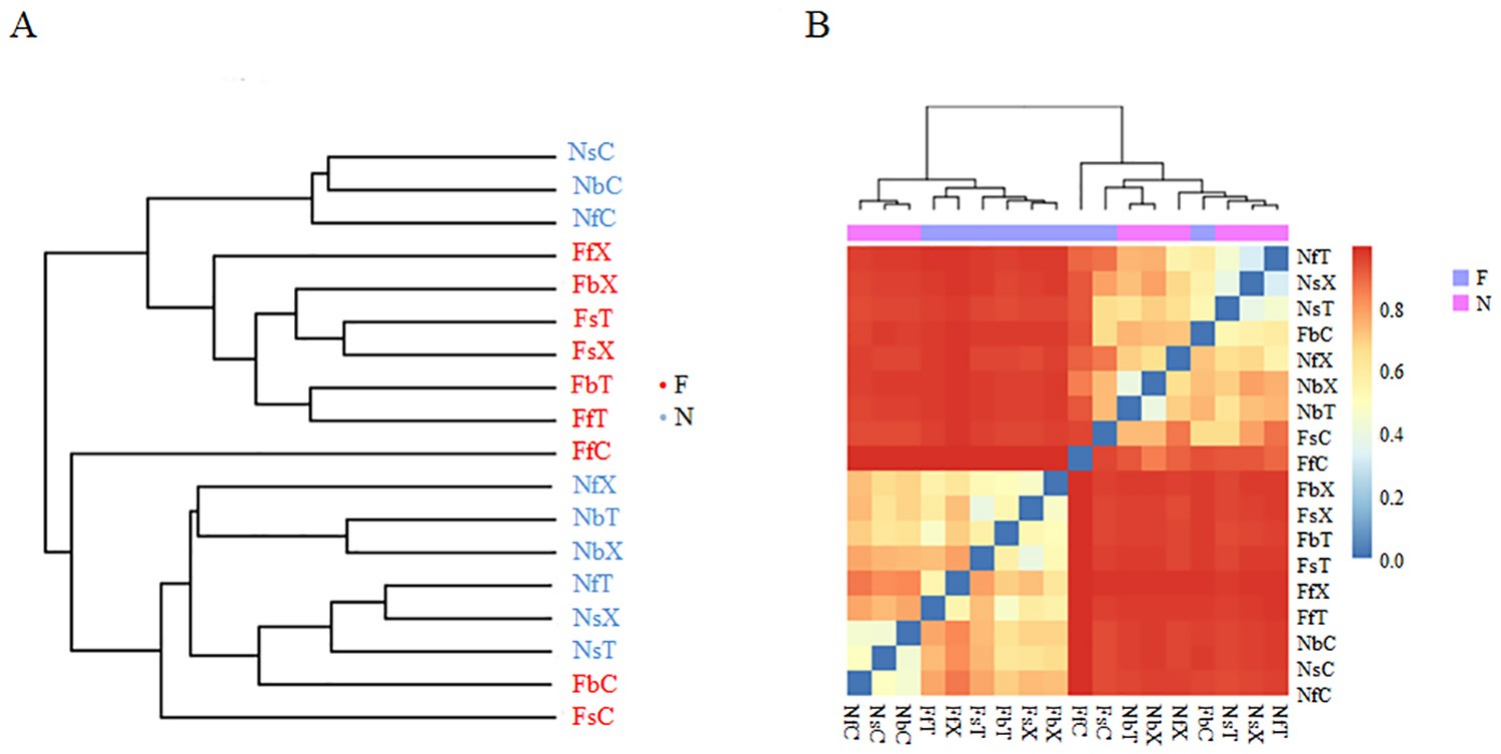
β-diversity analysis of the different treatments. A: Cluster analysis of the different treatments. B: Bray-Curtis distance analysis of the different treatments.

We analyzed the β-diversity of the samples based on a Bray-Curtis dissimilarity analysis. A cluster analysis indicated that samples from the same soil resources were clustered into one group (Fig 3A). The β-diversity of the different soils (mean Bray-Curtis: 0.97) was significantly higher than the β-diversity of the different developmental stages (mean Bray-Curtis N: 0.66, F: 0.60) (*P* < 0.01; S11 Table; Fig 3B). Statistical analyses were conducted to assess the contribution of each factor to the structure of the fungal community in the cotton rhizosphere, and the results indicated that species-level soil factors contributed approximately 42.27% to the fungal community structure in the cotton rhizosphere, which was higher than the other factors (*P* < 0.05; S11 Table).

## Discussion

### Difference in fungal community structure between the rhizosphere and bulk soil of cotton

Plant roots have a remarkable effect on the physical and chemical characteristics of soil, such as its structure and water retention [36–38]. The physical and chemical characteristics of root-associated soil are important because they determine both the physiological aspects of the root functions, such as water and nutrient uptake, and they influence microbial activity relevant to root growth [39–41]. Plant roots also release root exudates, volatile substances, border cells, and polymers into the soil environment and regulate the community structure of the rhizosphere microbiome through complex interactions with soil microorganisms [42–48], thereby promoting the colonization of beneficial microorganisms and inhibiting the colonization of harmful microorganisms [49]. Many studies have confirmed the existence of differences in the microbial communities of rhizosphere soil and the surrounding bulk soil of *Arabidopsis*, rice and *Populus* [6, 33, 50].

In the present study, the dominant fungal phyla in the rhizosphere of the two cultivars of cultivated allotetraploid *Gossypium* species were Ascomycota, Basidiomycota, and Zygomycota, which is consistent with that in the bulk soils. The relative abundance of each phylum in rhizosphere soil differed from that in the bulk soil to different degrees. The fungal communities influenced by cotton roots were mainly distributed in Basidiomycota. The dominant orders of Ascomycota and Zygomycota were consistent in the rhizosphere and bulk soils, whereas the dominant orders of Basidiomycota differed. In the rhizosphere soil, the dominant orders of Basidiomycota were Agaricales and Auriculariales in the FS and Agaricales and Trechisporales in the NS, which differed from that of the bulk soil. Thus, we speculate that the soil-derived fungal community composition determined the rhizosphere fungal community of cotton, whereas the cotton root affected the soil fungal community composition to a large extent. The β-diversity analysis and contribution analysis of each factor based on the Bray-Curtis dissimilarity confirmed that the soil resource in this study is the main factor that determines the rhizosphere fungal community.

### Rhizosphere fungal communities varied in the FS and NS

The characteristic of the soil itself is an important factor that affects the community structure of the plant rhizosphere microorganisms. Moreover, the microorganism composition of soil is the main cause of variation in the community structure of the rhizosphere microbiome [51, 52]. In this study, significant differences were presented in the rhizosphere fungal communities between different sources of soil. The difference was presented in two aspects: 1) The influences of cotton roots on the different fungal species were different. For example, in the NS, the relative abundance of *Engyodontium*, *Mortierella*, and *Penicillium* was lower in the pots containing cotton plants, whereas the relative abundance of *Clitopilus*, *Fusarium*, and *Rhodosporidium* was higher in the pots containing cotton plants. 2) The influence of cotton roots on some fungal communities differed substantially between the NS and FS soil. For example, the relative abundance of *Mrakia*, *Rhodosporidium*, and *Talaromyces* in the rhizosphere soil was higher in the NS but lower in the FS compared with that in the bulk soil. This difference might be attributed to the different characteristics of the two soil resources. Thus, we concluded that the cotton rhizosphere fungal community structure variation was mainly determined by the interaction of cotton roots with the different sources of soil.

Microbial diversity in soil is one of the major components determining soil health [28], and it is believed to be one of the main drivers in disease suppression [28–31]. Rhizosphere microbial diversity can improve a plant’s resistance to soil-borne disease [23]. Previous studies have shown that continuous cropping can decrease the structural and functional diversity of the soil microbiome [53, 54]. In agricultural production, soil quality degradation and aggravated plant diseases were the main reason that caused crop yield reduction in continuous cropped soil [55–57]. The fundamental reason for continuous cropping obstacles is related to disorders or deterioration of rhizosphere microorganisms (including rhizosphere fungi) [58, 59]. Bacterial diversity was presented in our previous study [34]. In the present study, the pots that did not contain plants had lower fungal α-diversity in the FS than in the NS, thus corroborating that long-term continuous cropping of cotton decreases fungal α-diversity, which in turn may be one of the important factors inducing continuous cotton-cropping obstacles. However, after planting with cotton, the fungal α-diversity of rhizosphere soils from the FS was increased compared with that in the bulk soil and higher than that of the NS. We speculate that fungal communities in continuously cotton-cropped FS might contain an abundance of fungi that are closely linked to cotton growth, nutrient absorption, and stress tolerance, and the functional limitation of such fungal communities is the main reason for continuous cotton-cropping obstacles.

### Developmental stages contributed to the variation of the fungal community in the cotton rhizosphere

Baudoin *et al*. proposed that the quantity and quality of root exudate input into the rhizosphere differ at different plant developmental stages, leading to differences in the composition of rhizosphere microbial communities between plant developmental stages [60]. Other studies have also demonstrated that rhizosphere microbes are significantly affected by the developmental stages of plants [61–65]. Our results indicated that the community composition of the cotton rhizosphere fungi varied significantly during different developmental stages. The species richness of the rhizosphere fungal communities was highest in the seedling stage in the FS and in the budding stage in the NS. In addition to the common dominant fungal genera of all three developmental stages, the rhizosphere fungal communities had a stage-specific dominant genus. The number of dominant genera was the highest in the budding stage, which may be related to the plant requiring specific materials or releasing certain hormones into the soil during this stage.

### EFG and DFG suggesting physiological characteristics of the cotton root

Rhizosphere microbial community structure could change plant physiological characteristics, for example, regulating plant growth and development [66], improve plant resistance to external stress [67], and improve plant nutrient absorption capacity [68]. And inversely, plant characteristics, for example, developmental stage [69], genotype [70], and nutritional absorption capacity [68] have great influence to plant rhizosphere fungal community structure, through root exudate chemical composition [71–73]. In the present study, changes of fungal genera varied between different treatments. Special fungal genera of genotype, developmental stages, and soil type suggesting special physiological characteristics of the cotton. *Penicillium* were promoted in FS which might attributed to the function of certain strains in *Penicillium*, for example, *Penicillium chrysogenum* [74], *Penicillium citrinum* [21], *Penicillium albidum* [75], and *Penicillium oxalicum* [76] were needed for cotton planting in continuous soil. *Preussia* was promoted in rhizosphere of TM-1 planting in FS, in seedling stage, which might attributed to the function of certain strains in *Preussia*, for example, *Preussia* sp. BSL-10 [77]. Most of reports about *Gibberella*, *Verticillium* were the pathogenicity to plants, such as *Gibberella zeae* [78], *Verticillium dahliae* [31]. In NS, *Gibberella* and *Verticillium* was inhibited in seedling and budding stage but promoted in rhizosphere of Hai 7124 in flowering stage, suggesting the inhabitation of cotton were decreased in flowering stage. In addition, EFGs and DFGs were differently TM-1 and Hai 7124 as well, which might be associated with the difference of species characteristics.

Our study provides insights into the structural variation of rhizosphere fungal communities under the influence of soil resources, developmental stage, and genotype, which might play key roles in cotton growth and health. The soil resources, cotton developmental stage, and cotton genotype all impacted the cotton rhizosphere fungal community composition. The composition of the cotton rhizosphere fungal community was primarily determined by the soil resources and regulated to a certain degree by the plant developmental stage and genotype.

## Supporting information

S1 Fig Rarefaction of samples based on the Simpson index. (TIF)

S2 Fig Rarefaction of samples based on the ACE index. (TIF)

S3 Fig Rarefaction of samples based on the Sobs index. (TIF)

S4 Fig Rarefaction of samples based on the Chao index. (TIF)

S5 Fig Rarefaction of samples based on the Shannon index. (TIF)

S6 Fig Relative abundance of fungal phyla in the bulk soil of both soils. (TIF)

S7 Fig Total number of OTUs of specific and common fungi in different treatments. (TIF)

S1 Materials and methods (DOC)

S1 Table Statistics and analyses of the sequencing data. (XLS)

S2 Table Relative abundance of fungi in the bulk soil. (XLS)

S3 Table Relative abundance of fungi in the rhizosphere soil. (XLS)

S4 Table Relative abundance increases in the rhizosphere fungal phyla compared with that in the bulk soils. (XLS)

S5 Table OTU numbers and α-diversity of each sample. (XLS)

S6 Table Fungal genera showing increases or decreases in relative abundance in the rhizosphere soil compared with the bulk soil in the field soil treatment. (XLS)

S7 Table Fungal genera showing increases or decreases in relative abundance in the rhizosphere soil compared with the bulk soil in the nutrient-rich soil treatment. (XLS)

S8 Table Relative abundance of fungal genera that were affected by the presence of cotton roots in the nutrient-rich soil. (XLS)

S9 Table Relative abundance of genera that were affected by the presence of cotton roots in the field soil. (XLS)

S10 Table EFG and DFG of TM-1 and Hai 7124 during different developmental stages in FS and NS. (XLS)

S11 Table β-diversity between samples. (XLS)
