# Supplementary figures and images for: Characterization and variation of the rhizosphere fungal community structure of cultivated tetraploid cotton
Source: PLoS One. 2019 Oct 18;14(10):e0207903. doi: 10.1371/journal.pone.0207903 (PMC6799950; doi:10.1371/journal.pone.0207903)

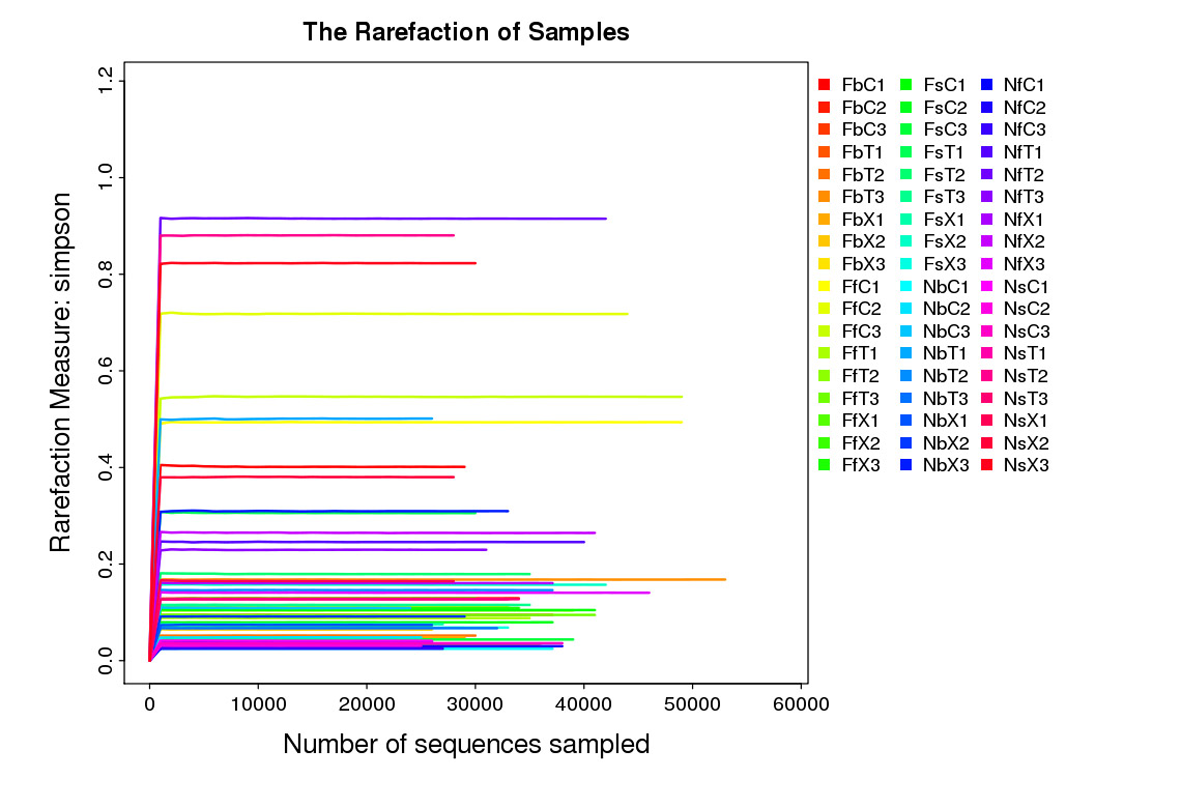

Supplement: S1 Fig — (TIF) [file pone.0207903.s001.tif]

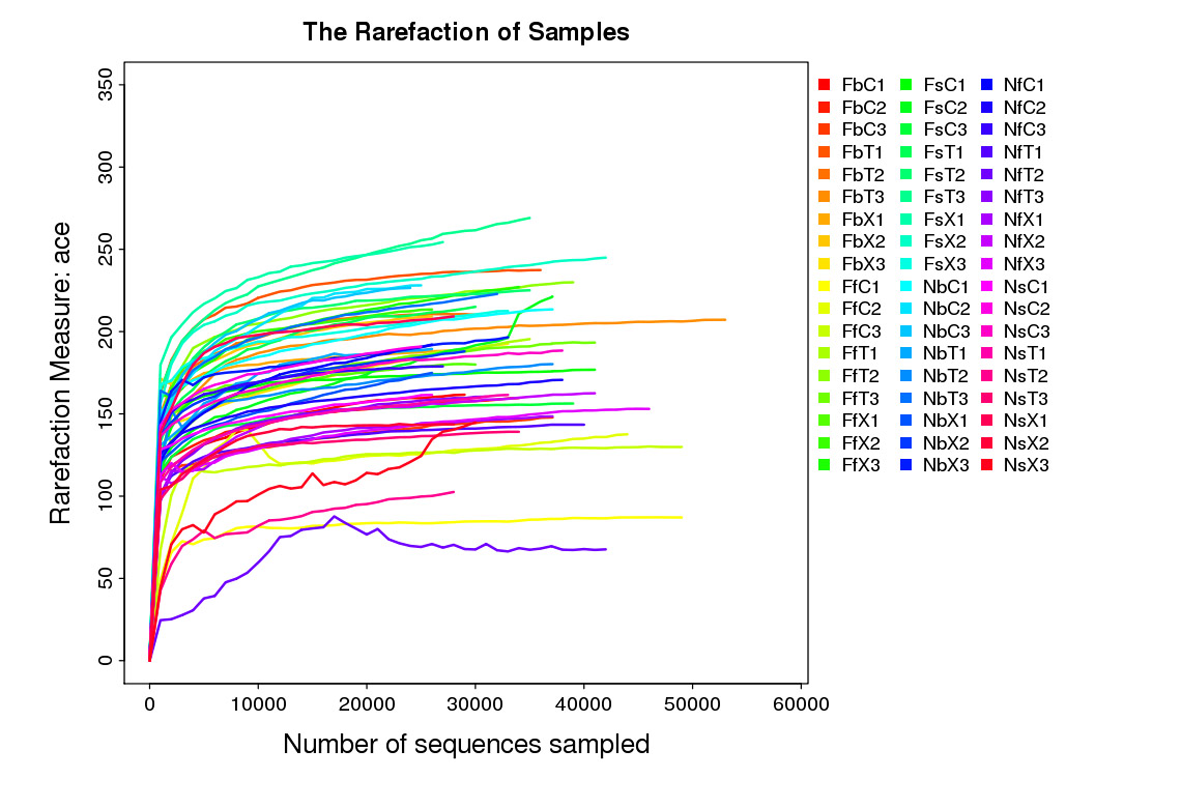

Supplement: S2 Fig — (TIF) [file pone.0207903.s002.tif]

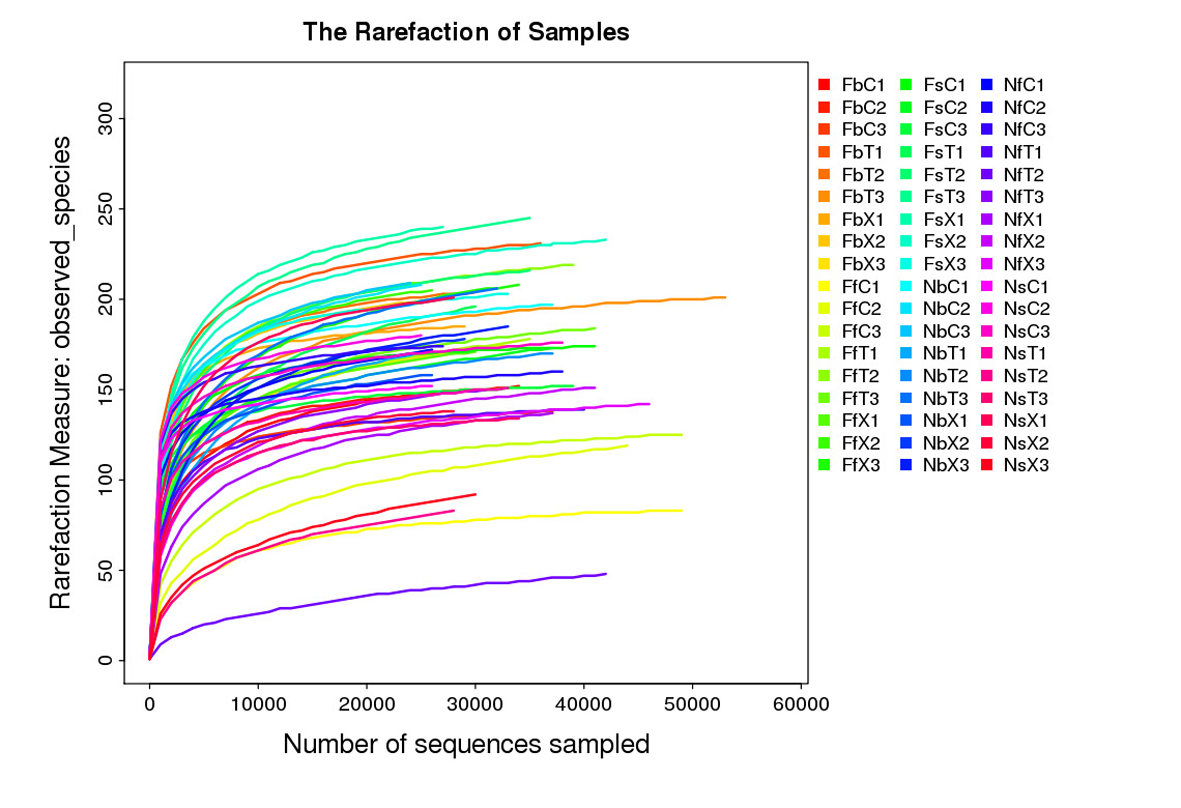

Supplement: S3 Fig — (TIF) [file pone.0207903.s003.tif]

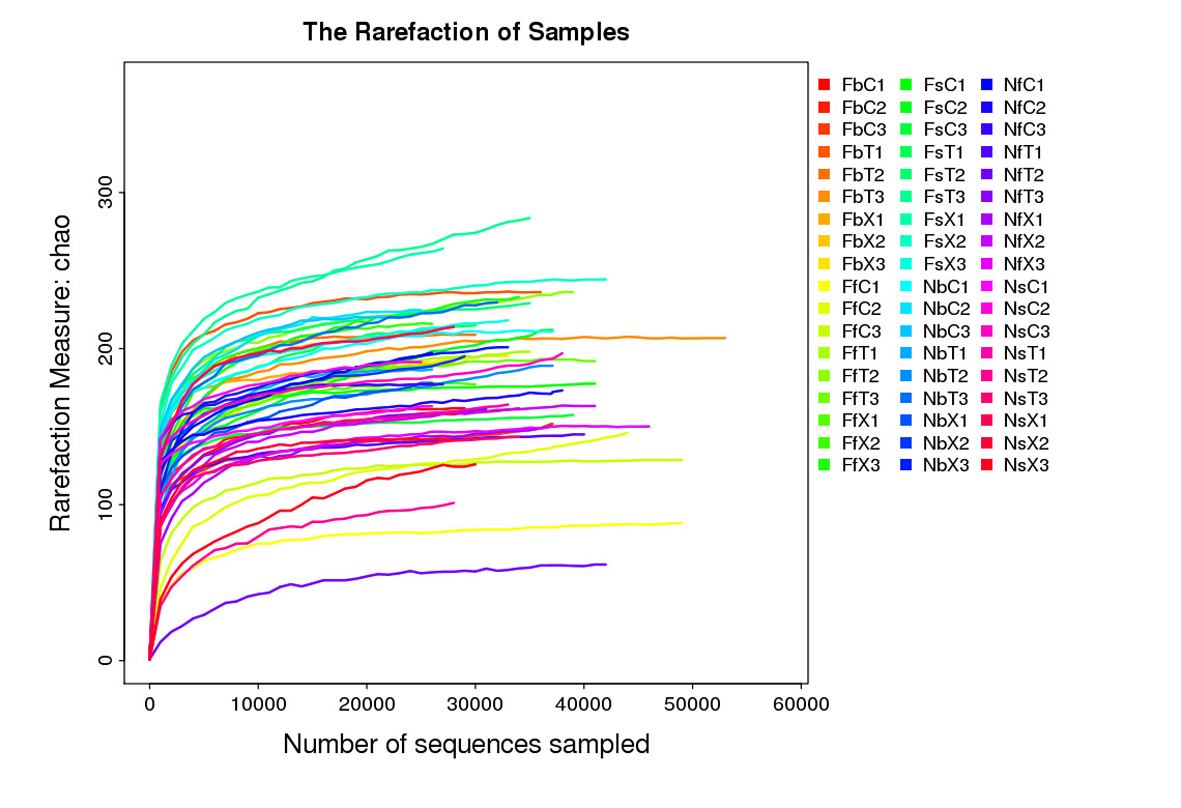

Supplement: S4 Fig — (TIF) [file pone.0207903.s004.tif]

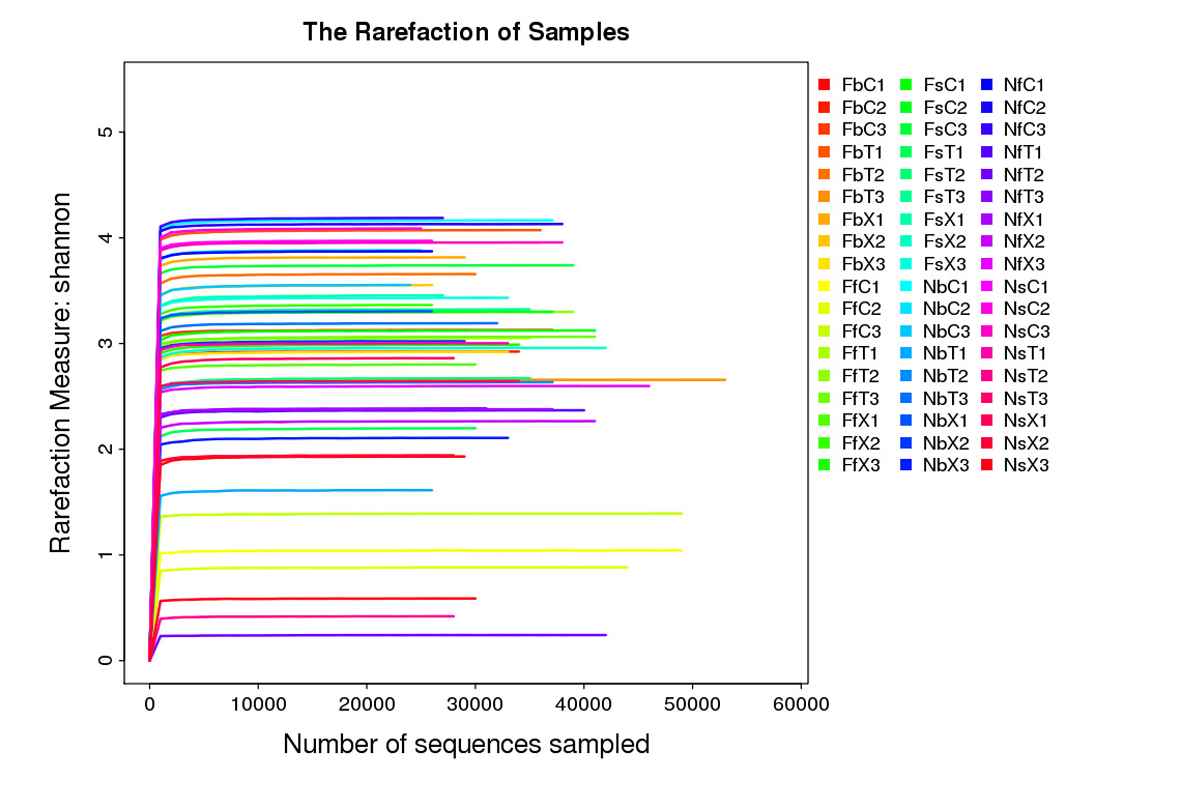

Supplement: S5 Fig — (TIF) [file pone.0207903.s005.tif]

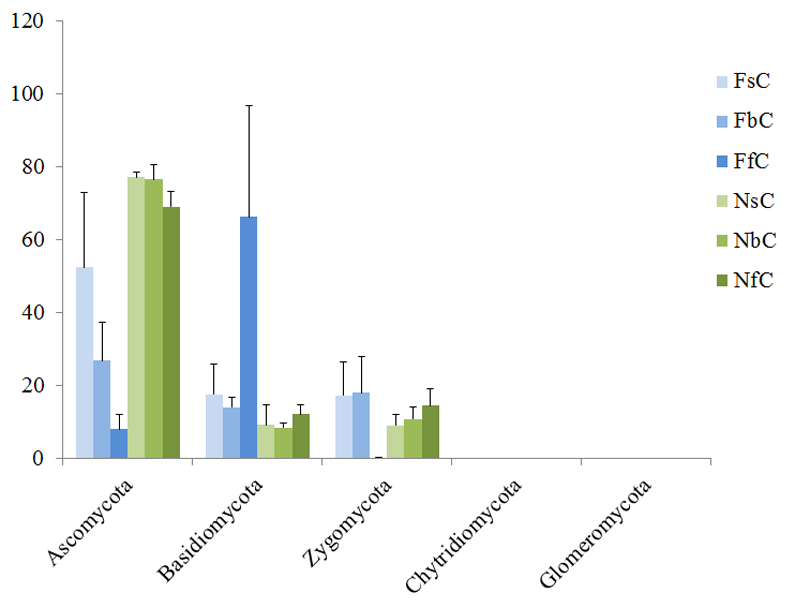

Supplement: S6 Fig — (TIF) [file pone.0207903.s006.tif]

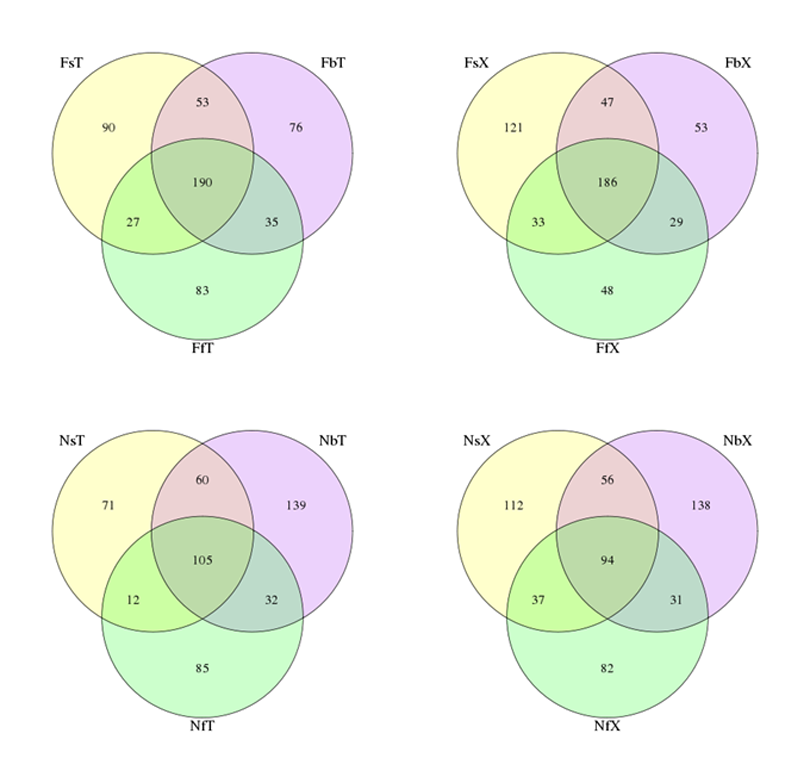

Supplement: S7 Fig — (TIF) [file pone.0207903.s007.tif]
