# Supplementary material for: Characterization and variation of the rhizosphere fungal community structure of cultivated tetraploid cotton
Source: PLoS One. 2019 Oct 18;14(10):e0207903. doi: 10.1371/journal.pone.0207903 (PMC6799950; doi:10.1371/journal.pone.0207903)
Supplement: S1 Materials and methods — (DOC) [file pone.0207903.s008.doc]

**Supplementary materials and methods**

**1. Test report of three different soils.**

| Code Of Sample | |  |  | test items | | | | | | | | | |
| --- | --- | --- | --- | --- | --- | --- | --- | --- | --- | --- | --- | --- | --- |
|  |  | hydrolysable nitrogen mg/kg | available phosphorus mg/kg | available potassium mg/kg | PH | organic material g/kg | exchangeable calcium g/kg | exchangeable magnesium g/kg | exchangeable sodium mg/kg | available cupper mg/kg | available zinc mg/kg | available iron mg/kg | available manganese mg/kg |
| 1 | N1 | 1458.06 | 176.68 | 1685.0 | 4.93 | 45.74 | 2.60 | 0.69 | 185.0 | 2.50 | 4.43 | 1222.46 | 238.51 |
| 2 | N2 | 1521.92 | 206.18 | 1685.0 | 4.77 | 40.62 | 2.43 | 0.68 | 182.0 | 2.81 | 4.45 | 1145.66 | 182.15 |
| 3 | N3 | 1191.99 | 182.24 | 1645.0 | 4.91 | 47.49 | 2.72 | 0.70 | 189.0 | 3.30 | 6.14 | 1245.26 | 247.27 |
| 4 | F1 | 35.22 | 19.35 | 84.0 | 8.53 | 10.25 | 6.89 | 0.62 | 87.0 | 1.36 | 2.26 | 2.99 | 1.78 |
| 5 | F2 | 36.45 | 20.63 | 85.0 | 8.53 | 10.62 | 6.70 | 0.57 | 89.0 | 1.49 | 2.31 | 3.56 | 1.77 |
| 6 | F3 | 35.63 | 20.97 | 82.0 | 8.48 | 10.50 | 6.46 | 0.54 | 89.0 | 1.36 | 2.21 | 3.09 | 1.68 |

Notes: F: field soil；N: nutrient-rich soil.

**2.** DNA information

| treament | sample | Concentration (ng/μL) | Volume (μL) |
| --- | --- | --- | --- |
| NfT | 1 | 135.2 | 45 |
|  | 2 | 102.7 | 45 |
|  | 3 | 125.7 | 45 |
| NfX | 1 | 165.8 | 45 |
|  | 2 | 100.8 | 45 |
|  | 3 | 164.5 | 45 |
| NbT | 1 | 72.7 | 45 |
|  | 2 | 103 | 45 |
|  | 3 | 91.7 | 45 |
| NbX | 1 | 108.1 | 45 |
|  | 2 | 86.5 | 45 |
|  | 3 | 43.2 | 45 |
| NsT | 1 | 56.6 | 45 |
|  | 2 | 166 | 45 |
|  | 3 | 96.7 | 45 |
| NsX | 1 | 105.8 | 45 |
|  | 2 | 125.6 | 45 |
|  | 3 | 129.5 | 45 |
| FfT | 1 | 44.3 | 45 |
|  | 2 | 1.4 | 45 |
|  | 3 | 21.1 | 45 |
| FfX | 1 | 81.9 | 45 |
|  | 2 | 136.4 | 45 |
|  | 3 | 81.4 | 45 |
| FbT | 1 | 20 | 45 |
|  | 2 | 32.3 | 45 |
|  | 3 | 33.5 | 45 |
| FbX | 1 | 10.1 | 45 |
|  | 2 | 48.5 | 45 |
|  | 3 | 40.1 | 45 |
| FsT | 1 | 52.6 | 45 |
|  | 2 | 30.6 | 45 |
|  | 3 | 17.6 | 45 |
| FsX | 1 | 26.2 | 45 |
|  | 2 | 37.5 | 45 |
|  | 3 | 31.4 | 45 |
| FfC | 1 | 58.7 | 45 |
|  | 2 | 62.7 | 45 |
|  | 3 | 48.7 | 45 |
| NfC | 1 | 9.9 | 45 |
|  | 2 | 9.4 | 45 |
|  | 3 | 6.1 | 45 |
| FbC | 1 | 91.7 | 45 |
|  | 2 | 68.5 | 45 |
|  | 3 | 55.3 | 45 |
| NbC | 1 | 14.3 | 45 |
|  | 2 | 13.5 | 45 |
|  | 3 | 7.4 | 45 |
| FsC | 1 | 61.3 | 45 |
|  | 2 | 40.6 | 45 |
|  | 3 | 69.2 | 45 |
| NsC | 1 | 11.6 | 45 |
|  | 2 | 9.4 | 45 |
|  | 3 | 13.3 | 45 |

**3. Data statistics:**

1. According to the phred algorithm, the raw data were filtered to over a 30 bp sliding window and truncated when average quality were lower than 20. The trimmed reads that having less than 75% of their original length were removed. Reads with ambiguous bases or reads with low complexity as well as their paired reads were removed.
2. The two overlapped high quality paired-end reads were combined to tags. The consensus sequence of paired end reads was generated by Fast Length Adjustment of Short reads, v1.2.11 with a minimal overlapping length of 15 bp and the mismatching ratio of overlapped region: ≤ 0.1. In order to ensure the quality of sequencing data, the quantity of tags ≥25,000.
3. Operational Taxonomic Units (OTUs) were clustered by tags using scripts of USEARCH software (v7.0.1090). Databases, UNITE(default): Version6 20140910[9], was used for species annotation.
4. Relative abundance : the number of the tags that clustered to the OTUs divided by the total number of the Tags. .
